# AlphaFold2 and its applications in the fields of biology and medicine

**DOI:** 10.1038/s41392-023-01381-z

**Published:** 2023-03-14

**Authors:** Zhenyu Yang, Xiaoxi Zeng, Yi Zhao, Runsheng Chen

**Affiliations:** 1 West China Biomedical Big Data Center, West China Hospital, Sichuan University, Chengdu, 610041 China; 2 Key Laboratory of Intelligent Information Processing, Advanced Computer Research Center, Institute of Computing Technology, Chinese Academy of Sciences, Beijing, 100190 China; 3 Key Laboratory of RNA Biology, Center for Big Data Research in Health, Institute of Biophysics, Chinese Academy of Sciences, Beijing, 100101 China; 4 Pingshan Translational Medicine Center, Shenzhen Bay Laboratory, Shenzhen, 518118 China

**Keywords:** Computational biology and bioinformatics, Structural biology

## Abstract

AlphaFold2 (AF2) is an artificial intelligence (AI) system developed by DeepMind that can predict three-dimensional (3D) structures of proteins from amino acid sequences with atomic-level accuracy. Protein structure prediction is one of the most challenging problems in computational biology and chemistry, and has puzzled scientists for 50 years. The advent of AF2 presents an unprecedented progress in protein structure prediction and has attracted much attention. Subsequent release of structures of more than 200 million proteins predicted by AF2 further aroused great enthusiasm in the science community, especially in the fields of biology and medicine. AF2 is thought to have a significant impact on structural biology and research areas that need protein structure information, such as drug discovery, protein design, prediction of protein function, et al. Though the time is not long since AF2 was developed, there are already quite a few application studies of AF2 in the fields of biology and medicine, with many of them having preliminarily proved the potential of AF2. To better understand AF2 and promote its applications, we will in this article summarize the principle and system architecture of AF2 as well as the recipe of its success, and particularly focus on reviewing its applications in the fields of biology and medicine. Limitations of current AF2 prediction will also be discussed.

## Introduction

In December of 2020, AlphaFold2 (AF2),^1^ a machine-learning based model to predict protein structures developed by DeepMind, won the championship in the 14th Critical Assessment of Structure prediction (CASP14).^2^ One and a half years later, DeepMind and the EMBL’s European Bioinformatics Institute (EMBL-EBI) released structures of more than 200 million proteins predicted by AF2,^3^ which cover almost all the known proteins on the planet (protein universe). These two events have drawn great attention to AF2 in the science community. AF2 represents a milestone advance in protein structure prediction. It is considered as the greatest contribution of artificial intelligence (AI) to the scientific field and one of the most important scientific breakthroughs made by mankind in the 21st century. This is a very remarkable historical achievement in the human understanding of nature. The high appraisal to AF2 is not excessive because understanding the three-dimensional (3D) structures of proteins is one of the most challenging issues in the field of biology, which has puzzled scientists for 50 years.^4^ Although multiple technologies including nuclear magnetic resonance (NMR),^5^ X-ray crystallography,^6^ and cryo-electron microscopy (cryo-EM)^7^ have been adopted to solve the protein structures, only about 200,000 proteins’ structures have been determined (https://www.rcsb.org/), covering less than 0.1% of the protein universe.

AF2 is expected to have a significant influence on the fields of biology and medicine, and may change the way we do related researches such as structural biology, drug discovery, protein design, etc. Despite that the time is short since AF2 was developed, there are already many studies related to AF2 reported. To better understand AF2 and promote its applications, we will in this review paper summarize the algorithm and working principle of AF2 and recipe of its success, particularly focus on reviewing its applications in the fields of biology and medicine. Limitations of current AF2 prediction will also be discussed. The remaining part of this paper is organized as follows. We will firstly give a brief introduction to the protein structure prediction, followed by analyzing the principle and architecture of AF2 and the secret of its success. Then we will summarize the applications of AF2 in the fields of biology and medicine, and discuss limitations of current AF2 prediction. It will end in concluding remarks.

### A brief introduction to the protein structure prediction

In 1961, Anfinsen^8^ raised the famous thermodynamic hypothesis of protein folding (“Anfinsen’s dogma”) that a protein’s native structure stands for a free energy minimum determined by its amino acid sequence, or in other words, the 3D structure of a protein is only determined by its amino acid sequence. This hypothesis is the theoretical foundation of protein structure prediction. Since then, people began to look for algorithms to directly predict 3D structures of proteins from amino acid sequences. In the field of protein structure prediction, CASP, founded in 1994, is a milestone event.^9–11^ This competition is held every two years. The CASP committee publishes the “target sequences” globally, for which the experimental structures are known but not yet released. Each participant team that registers for the competition will predict and submit structures of proteins corresponding to the “target sequences” by using their own algorithm within a specified period. Finally, the CASP committee will assess their predicted structures by comparing with those experimentally solved. The competition is double blinded: participants have no access to the experimental structures and referees do not know who make the submissions. Because of the objectivity and fairness, the CASP competition has a very high reputation in structural biology and computational biology communities.

Until now, many algorithms for protein structure prediction have been reported and readers can refer to several recent review papers.^12–17^ Despite vastly different, they can be roughly grouped into three major classes: homology modeling, de novo modeling, and machine learning (ML) -based modeling.

**(1) Homology modeling**

Homology modeling, also known as comparative modeling or template-based modeling, is based on the hypothesis that proteins’ 3D structures are more conserved than their amino acid sequences, and that therefore similar amino acid sequences should have similar 3D structures.^18,19^ The homology modeling method mainly uses two techniques: sequence alignment and molecular modeling. The basic workflow of homology modeling is as follows: Given a target amino acid sequence, the first step is to look for its homologous sequences from structure-known protein databases, followed by sequence alignment. Then, coordinates of amino acids of the structure-known homologous proteins are taken as the coordinates of the corresponding amino acids of the target protein. Subsequently, molecular modeling is performed to relax the unfavorable interactions between amino acid pairs. Finally, the generated 3D structure is evaluated.

The homology modeling method is the most popular approach decades ago.^19–24^ Advantages of the homology modeling include simple algorithm, fast prediction speed, and high accuracy for proteins that have structure-known homologs. The defect is that it strongly depends on the template structures, which means that it cannot predict structures of proteins whose homologs’ structures have not been determined.^25^

**(2) De novo modeling**

De novo modeling is a protein structure prediction method based on the “first principles”.^26^ Unlike the homology modeling, the de novo modeling does not depend on the known protein structures, but generating the 3D structure of a target protein only based on the established laws of physics (quantum mechanics). In brief, a de novo modeling method conducts conformation search guided by a designed energy function with the atomic coordinates of amino acids as variables. Many possible conformations are produced in this process and that with the lowest energy is picked. Obviously, the de novo modeling method depends on two factors: (1) an energy function that represents the free energy of target protein with respect to the atomic coordinates of amino acids; (2) an effective conformational search algorithm that can quickly identify low energy states.

There are many investigations regarding protein structure prediction based on de novo modeling.^27–33^ The advantages of de novo modeling include: (1) it does not rely on the known protein structures, which means that it is able to predict protein structures where no any prior structural knowledge exists; (2) it has the possibility of finding new protein structural types. Nevertheless, this method faces two major obstacles. The first one is the free energy function. Theoretically, accurate calculation of free energy needs to solve the Schrödinger’s equation, which requires huge amount of calculation that we cannot afford even now. Therefore, empirical formulae have to be used. Currently, a majority of empirical formulae are based on molecular mechanics or Newtonian mechanics. The second one is the conformational space of protein, which is an astronomical number. The possible conformational number of a protein with several hundred amino acids is estimated to be about 10^300^.^34^ Although great progresses have been made in conformational search algorithms, as well as computing power and storage space, de novo modeling is still only applicable to small proteins with the number of amino acid residues ranging from 10 to 80.

**(3) ML-based modeling**

ML-based modeling is a strategy that utilizes ML algorithms and known protein structures to predict the structures of target proteins. Despite many ML algorithms, the most noteworthy is deep learning (DL). DL has achieved rapid development in recent years, which was driven by the fast growing of data volume (“big data”), a large increase in computing power (e.g., GPU, TPU, etc.) and the continuous optimization of DL algorithms (e.g., Recurrent Neural Networks,^35^ Convolutional Neural Networks,^36^ Generative Adversarial Networks,^37^ Transformer,^38^ etc.). DL has demonstrated its great power in computer vision,^39^ natural language processing,^40^ auto-driving,^41^ and other fields.^42–46^ Recently, DL has also been applied to the protein structure prediction.^12^ At present, there are many modeling methods based on DL, among which AlphaFold,^1,47^ RoseTTAFold,^48^ ESMFold^49^ (ESMFold also offers an extensive database of protein structural predictions, which include 617 million metagenomic protein structures) and the recent language model by Chowdhury et al.^50^ are the most famous ones.

**Fig. 1:**
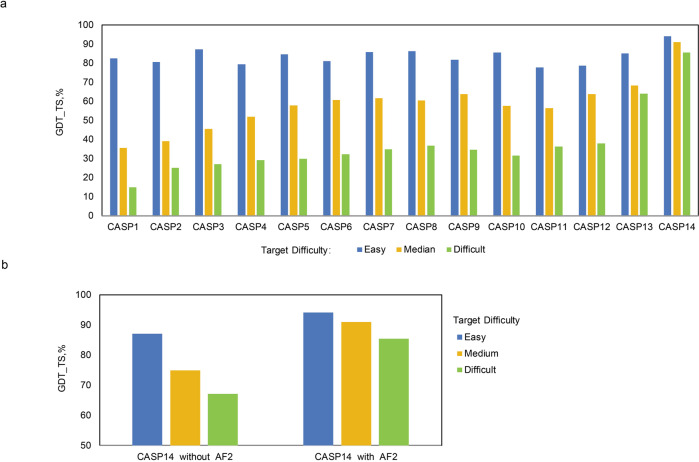
Performances of protein structure prediction indicated as backbone agreement with that of structures determined by experiments for the best models in CASPs. **a** The trend of performance (denotated by GDT_TS values) with regard to the backbone accuracy for best models obtained in each CASP. **b** A comparison of the GDT_TS values with or without AF2 prediction included in CASP14. Prediction accuracies for proteins with different target difficulty (“easy”, “medium” and “difficult”) are presented in indicated colors (blue, gold and green, respectively)

Compared with homology modeling and de novo modeling, the DL-based method is a data-driving approach and is the latest emerging one. Due to the great success of DL in other fields, the DL-based protein prediction approach is expected to have a better performance. Indeed, the DL-based method lived up to people’s expectation and won the champion of CASP13^51^ and 14.^2^ Particularly, AF2 in CASP14 could predict the structures of proteins with atomic-level accuracy. Figure 1a summarizes the trend of performance denoted as the backbone accuracy for the best models obtained in each CASP.^52^ Here the backbone accuracy is measured by the Global Distance Test (GDT_TS)^53^ value, which is a multi-scale metric to indicate the proximity of the Cα atoms in a model to those in the corresponding structure determined by experiments. The GDT_TS values were calculated respectively according to proteins with different target difficulties: “easy”, “medium”, and “difficult”; an “easy” target implies a protein whose structure is easy to be predicted, for example, a well-folded protein with no loop and structures of its highly homologous proteins being available, while a “difficult” target implies a protein whose structure is difficult to be predicted, for example, a protein with some un-folded domains or many loops, and structures of its homologous proteins being not available. As shown in Fig. 1a, the “easy” proteins can be predicted accurately in CASP1 - CASP14 with GDT_TS values around or larger than 80%. However, for the “medium” and “difficult” proteins, the prediction accuracies were significantly improved only in CASP13 and CASP14. Especially, the GDT_TS values for the “medium” and “difficult” proteins have reached more than 85% in CASP14, largely due to the contribution of AF2; Fig. 1b shows a comparison of the GDT_TS values with or without AF2 prediction included in CASP14. We will elaborate the principle and architecture of AF2 as well as the secret of AF2’ success in the next section.

### Principle and architecture of AF2 and secrets of AF2’ success

AF2 is the most advanced protein structure prediction method of DeepMind. Its principle is based on the state-of-the-art DL algorithms as well as the conservation of protein structures in evolution. It uses a new end-to-end deep neural network which is trained to generate protein structures from amino acid sequences, by utilizing information of homologous proteins and multiple sequence alignments.

In AF2, some new DL algorithms developed recently are used, of which attention mechanism-based transformer^38^ plays a critical role in improving AF2’s performance. Transformer is a newly emerging deep neural network, which applies the self-attention mechanism to obtain intrinsic features and displays great potential of broad applications in AI. Transformer^38^ was first applied in the area of natural language processing (NLP). It is composed of an encoder module and a decoder module with several transformer blocks of the same architecture. Each transformer block is composed of a multi-head attention layer, a feed-forward neural network, shortcut connection and layer normalization.

The conservation of protein structure in evolution is the biological principle behind AF2. A protein is often conservative in evolution, and the evolution is mostly neutral, which means that most of the mutations don’t affect the protein function. More importantly, protein structure is more conservative than its amino acid sequence. Typically, for example, for a sequence that change by 80% between distant species, the 3D structure may remain almost the same. Conservation of a position in alignment usually implies its importance for protein folding or function. Co-evolution of two amino acid residues of a protein often implies interaction between those amino acids. This information has been used as the basis for 3D structure prediction in AF2.

**Fig. 2:**
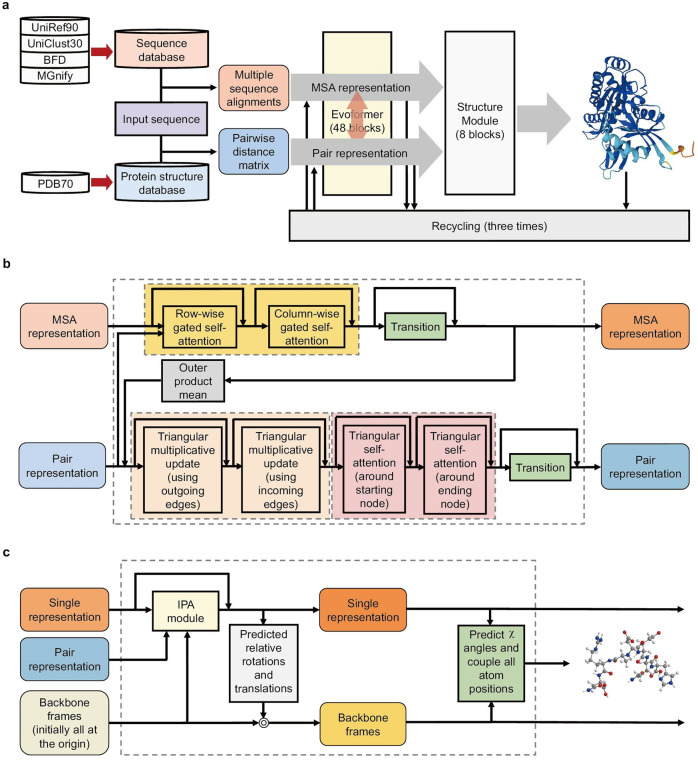
Schematic work principle and architecture of AF2. **a** The overall architecture of AF2. The pipeline of AF2 contains three modules. The first one is the input module, which takes an amino acid sequence as input, and generates the MSA representation and the pair representation. The second one is the Evoformer module, which takes the MSA representation and the pair representation from the first module and passes them through the deep learning module, Evoformer. The third one is the structure module, which achieves the transition from abstract representation of protein structure to 3D atom coordinates of target protein. **b** Components of a block in Evoformer. Evoformer contains 48 blocks with weights not shared. The MSA representation and the pair representation are renewed through each block. **c** Components of a block in the structure module. Structure module contains 8 blocks with shared weights. Single representation and backbone frames are updated through each block of the structure module

AF2 adopts an architecture that is completely different from that of previous DL models, including AlphaFold1.^47^ A detailed description for the architecture of AF2 can be found in reference.^1^ Here we present an overview to the architecture and work principle of AF2. As shown in Fig. 2, the pipeline of AF2 includes three modules.

1. The first one is the input module. Given an amino acid sequence, AF2 finds its homologs in sequence databases and conducts MSA by aligning the input sequence and its homolog sequences. AF2 also checks whether any of the homologs has a 3D structure available in protein structure databases, and constructs a pairwise distance matrix between amino acids. Then AF2 generates MSA representation and pair representation. It should be noted that, although both AF2 and homology modeling use MSA, AF2 extracts and utilizes the co-evolution information from the MSA, but homology modeling does not. Intuitively, when two residues (A and B) are spatially near to each other in the folded structure, the mutations of residue A may provoke a selective pressure for residue B to mutate. Such co-evolutionary information^54^ detected in MSAs has been utilized to assist the protein structure prediction in AF2.
  It is necessary to mention that AF2 uses many high-quality protein sequence databases, including Uniref90,^55^ Uniclust30,^56^ MGnify^57^ and BFD (Big Fantastic Database);^1^ BFD is a database constructed by the team themselves. Pertaining to the structure databases used for training and as templates, it adopts PDB and PDB70^58^ respectively. AF2 also utilizes several efficient search algorithms, including JackHMMER^59^ and HHBlits^60^ for genetic searching, and HHSearch^61^ for template searching.
2. The second one is the Evoformer module, which is likely an encoder. In this module, AF2 takes the inputs (MSA representation and pair representation) from the first module and passes them through a deep learning module (called Evoformer). Evoformer produces processed MSA representation and pair representation. The key benefit of using Evoformer blocks is that they are able to switch information between MSA representation and pair representation: the MSA information can be reinterpreted as the pairwise information is improved, and in the similar way, the pairwise information can be further improved as the MSA information is reinterpreted.
  The Evoformer contains 48 blocks with weights not shared. Each block has two inputs: an MSA representation and a pair representation. The outputs from each Evoformer block are an updated MSA representation and an updated pair representation (Fig. 2b). The MSA representation and pair representation are processed with several layers. The Dropout approach is also used, which is commonly used for alleviating the problem of overfitting.
  Each Evoformer block (Fig. 2b) contains two pathways of transformer-based layers and two “communication channels” between the two pathways. The first pathway of transformer-based layers acts on the MSA. It computes attention over a large matrix of protein symbols. To reduce computational cost, the MSA attention is factorized in row-wise gated self-attention and column-wise gated self-attention components. The row-wise gated self-attention mechanism, allowing the network to identify which pairs of amino acids are more related, constructs attention weights for amino acid pairs. It also combines the information from the input pair representation, and this information can be considered as an extra term. The column-wise gated self-attention, allowing the network to determine which sequences are more informative, enables the components which belong to the same target amino acid to process information exchange. After the row-wise gated self-attention and column-wise gated self-attention steps, the MSA pathway has an MSA transition layer which includes a 2-layer MLP. This trick enhances the attention mechanism and allows it to pinpoint interacting pairs of amino acids.
  The second pathway of transformer-based layers acts on the pair representation. The key feature of this network is that attention is arranged in terms of triangles of residues, which is based on the straightforward principle that in a triangle, any two edges can affect the third edge. The intuition here is to enforce the triangle equivariance. As shown in Fig. 2b, the first two rounds of update are triangular multiplicative updates, which are based on non-attention method. Each of the “outgoing” and “incoming” edges obtains an update from another two edges of all the triangles where the edge is included. The second two rounds of update are triangular self-attention. They update the pair representation in the Evoformer block. Two versions are also involved: “starting node” version and “ending node” version. The “starting node” version renews the edge based on all the edges which has the same starting node. The “ending node” version operates the similar way, but it works on the edges which share the same ending node instead. Pairwise representation pathway also contains a transition layer after the triangular self-attention layers, which works the same way as the transition layer introduced above.
3. The third one is the structure module, likely a decoder. The structure module also uses a transformer neural network. It achieves the transition from abstract representation of protein structure to 3D atom coordinates of target proteins. The structure module takes each residue as a separate object and predicts the rotations and translations required to place it.

The structure module has three input elements. The first one is the single representation containing the abstract information of the target sequence. It is a linear projection of the MSA representation’s first row. The second one is the pair representation output from the Evoformer module. Backbone frames serve as an additional input. Each residue is represented as a triangle, where its vertex next to obtuse angle is Cα atom, and two other vertices are N atom of amino group and C atom of carbonic acid group. The backbone frames, which are the main part of the system prediction, are formed with triangles of the whole amino acid sequence. At the beginning of the structure module, all the backbone frames are placed at the same point in the same orientation. The structural module’s output is the 3D coordinates of all the protein atoms.

The structure module has 8 blocks with shared weight. Each block (Fig. 2c) updates the single representation and the backbone frames. The critical component of each block is the Invariant Point Attention (IPA), which is a geometry-aware attention mechanism used for updating the single representation. The final attention values of the IPA operation are 3D equivariant, which means that they are invariant to global rigid motion including rotations and translations. After the processing of the IPA operation, the module block predicts relative rotations and translations of each backbone frame. The utilization of these operations enables the overall attention and process equivariantly on the backbone frames. In the next step, the structure module block predicts the side-chain χ angles and computes all atom positions using the updated single representation from IPA and the renewed backbone frames. However, the final output might not meet all the stereochemical constraints. For this reason, AF2 applies Amber relaxation to resolve the violations and clashes without harmfully impact the prediction accuracy. OpenMM^62^ with Amber99sb force field^63^ is used for the process.

Finally, AF2 adopts recycling mechanism for three times to process iterative refinement of training and testing; the recycling mechanism has been broadly utilized in computer vision, which allows the network to be deeper and to process multiple versions of the input features without significantly increasing the quantity of parameters or training time. In each recycling, the model incorporates the previous outputs as additional inputs. AF2 recycles the predicted backbone atom coordinates from the structure module, the output pair representations and the first row of MSA representations from the Evoformer.

AF2 has achieved the best performance compared to previous models. Although we have presented the principle and architecture of AF2, the secret of AF2’ success is not explicitly indicated. Here, we present our analysis on the most critical points leading to the success of AF2. From the technological point of view, it is indisputable that the delicate algorithms used are the major causes. Of the most importance is the use of attention mechanism-based transformer. In AF2, several types of attention mechanisms are used, with each one focusing on a specific aspect for the model to learn. In the encoder part, AF2 uses two groups of transformers which are intertwined with each other: one mainly operates on the raw MSA and the other one mainly operates on pairwise information, which update each other through specific information channels between them. The MSA row-wise gated self-attention allows the model to capture long-range dependencies in amino acid sequences and protein structures. The MSA column-wise gated self-attention is a kind of ‘conservation-aware’ attention mechanism, which lets the elements exchange information among species. The triangular self-attention module in the decoder enables the model to learn geometric restrictions within the protein molecules. In the decoder part, AF2 also employs a transformer to geometrically encode residues as a cloud of oriented reference frames in 3D space.

The training method is also a factor which makes AF2 success. The designers utilized the idea of self-distillation.^64^ They used a combination of PDB and a new self-distillation unlabeled data set of predicted protein structures as the training data to train AF2, among which, 25% of the training example comes from the known structures in PDB while 75% of data was from the new self-distillation data set. The aim is to make AF2 recap the protein structures predicted previously challenging by using different training data augmentation methods. This integration data set approach makes use of the data predicted by AF2 and largely improves the performance of the model.

Other algorithms or tricks that may contribute to the success of AF2 include the use of recycling approach, end-to-end framework for learning from protein data, and so on. Moreover, big data of amino acid sequences and structures also contribute a lot to AF2’s success. The complete sequence library and sufficient number of single domain protein structures allow deep learning neural networks to explore various dependencies in protein sequence and structure, which could be another important intrinsic cause for the success of AF2.

### Applications of AF2 in the fields of biology and medicine

**Fig. 3:**
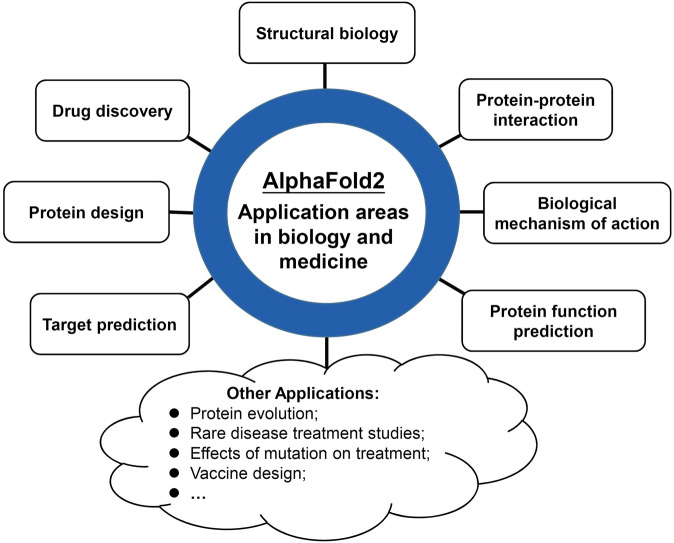
Application areas of AF2 in the fields of biology and medicine. AF2 can be applied in many areas of biology and medicine, including structural biology, drug discovery, protein design, protein-protein interaction, target prediction, protein function prediction, biological mechanism of action, and others (such as protein evolution, rare disease treatment studies, effects of mutation on treatment, vaccine design and so on)

The excellent performance of protein structure prediction by AF2 and the release of structures of more than 200 million proteins are reshaping structural biology, and hence will profoundly impact the fields of biology and medicine that require protein structural information. AF2 and its predicted protein structures will enable researchers to have more opportunity to solve problems that are previously thought to be highly challenging. We will in the follows review the progress of applications of AF2 in the fields of biology and medicine. These applications are classified into eight categories: structural biology, drug discovery, protein design, target prediction, protein function prediction, protein-protein interaction, biological mechanism of action, and others (Fig. 3).

#### Structural biology

Undoubtedly, structural biology is the most impacted area by AF2.^65^ Rather than saying that AF2 may make structural biologists unemployed, we prefer to the viewpoint that the AF2 and its predicted structures will change the way we do structural biology, including X-ray crystallography, cryo-EM, and NMR spectroscopy. Firstly, predicted structures could be utilized as templates for molecular replacement in solving X-ray crystal structures, implying that traditional selenomethionine phasing is almost not necessary.^66,67^ Secondly, these predicted structures may also be helpful for structure determination of large protein assemblies by cryo-EM, which usually needs structures of the component proteins or their domains as a starting point for fitting to the cryo-EM densities. Thirdly, one could also benefit from the predicted structures in using NMR to solve protein structures.^68,69^ Typically, the de novo structure determination of domains or proteins using NMR, which is time-consuming, may be replaced by the AF2 structures. Therefore, the application of AF2 prediction allows to make full use of the advantages of NMR in studying protein folding and dynamics.

Currently there are already many successful applications in this respect. For example, Hu et al.^70^ utilized X-ray crystallography and AF2 prediction to determine the structure of the VP8* domain (VP8*B) of VP4, which is a spike protein, in group B rotaviruses. In this study, the authors expressed and purified the VP8*B protein. Then they obtained the crystals of this protein and received diffraction data of X-ray. In the process of solving the 3D structure of this protein, instead of using the traditional selenomethionine phasing method, they used AF2 to generate a suitable search model for molecular replacement. The results showed that the AF2 predicted structure almost perfectly matched the diffraction density. Besides the overall fold, AF2 also successfully predicted the orientation of sidechain with high accuracy, which is very close to that determined by experiments. Of note is that they found a novel fold mode by AF2, which has never been reported in homology proteins.

Hutin et al.^71^ recently revealed a structure of the vaccinia virus DNA helicase, the helicase-primase D5, by utilizing combined cryo-EM and AF2 prediction. The obtained structure of D5 shows an AAA + helicase core, which is flanked by N- as well as C-terminal domains. The structure of D5 predicted by AF2 largely helped the construction of the model. The N-terminal domain, which has a 3.9 Å resolution, forms a well-defined tight ring, while the resolution decreases towards the C-terminus, which still allows the fit of the predicted structure. This structure validates AF2 calculations of a large number of structures of viral helicase associated with D5. Jin et al.^72^ solved the structure of interleukin (IL) −27 signal complex by using cryo-EM with the aid of AF2 prediction, through which they revealed a new mechanism for the assembly and activation of IL-27 receptor recognition complex. Skalidis et al.^73^ utilized cryo-EM and AF2 to characterize metabolon-embedded architectures of a 60S pre-ribosome, fatty acid synthase, and pyruvate/oxoglutarate dehydrogenase complex E2 cores. Though cryo-EM 3D reconstructions were resolved at resolution ranging from 3.84 to 4.52 Å by collecting less than 3,000 micrographs of a single cellular fraction, AF2 enabled polypeptide hydrogen bonding patterns discernible at this resolution range. These results proposed an integrated approach, powered by ML, which enables the cryo-EM characterization of native cell extracts.

Fowler and Williamson^68^ recently evaluated the accuracy of NMR structures and AF2 prediction. They used the program Accuracy of NMR Structures Using RCI and Rigidity (ANSURR), which calculates a protein structure’s local rigidity.^74^ They compared AF2 predicted structures and those determined by NMR and found that AF2 tends to be more accurate than NMR ensembles. They also found that the NMR ensembles are more accurate in some cases, which tend to be dynamic structures, where AF2 had low confidence. They finally suggested that AF2 could be utilized as the model for refining NMR-structure.

There are also some other similar studies in which AF2 is applied to help structural determination, and some of which combines AF2 and experimental methods to verify if the protein structure is solved correctly, for example, combining AF2 with X-ray crystallography,^75,76^ cryo-EM,^77–79^ NMR,^80^ and multiple methods.^81–85^

Besides structure determination, AF2 prediction can even be applied to the design of expression constructs. They enable researchers to better determine where the starting and ending points of a domain locate in the sequence, and avoid less ordered regions;^86^ Neglecting less ordered regions from protein sequences is often conducive to designing recombinant proteins for investigations pertaining to structures.

#### Drug discovery

Drug discovery is one of the major application areas that require protein structure information. Although the confidence level of prediction varies, the AF2 predicted structures still could considerably promote the structure-based drug discovery, especially against protein targets with limited or no structural information. At present, protein structures used in structure-based drug discovery mainly come from the RCSB Protein Data Bank (PDB). However, the number of protein structures in the PDB database are quite limited, which is far from meeting the current vigorous demand for drug discovery. The structures’ release of the entire protein universe is expected to accelerate existing and new drug discovery projects.

Zhang et al.^87^ recently used Glide,^88^ a molecular docking program, to benchmark the performance of virtual screening towards 28 common drug targets, each with a known experimental structure and an AF2 structure. The AF2 structures show comparable performance with experimental structures in terms of the enrichment factor, especially when flexible docking was used. The results clearly show that AF2 structures can completely replace the experimental structures in virtual screening.

Ren et al.^89^ applied AF2 in their end-to-end AI-powered drug discovery engines, which include a biocomputational platform named PandaOmics and a generative chemistry platform named Chemistry42.^90^ PandaOmics provides the targets of interest and Chemistry42 is responsible for generating molecules based on the AF2 predicted structures, and the selected molecules are then synthesized and tested in biological assays. Through this approach, they discovered a small molecule hit compound for CDK20 (Cyclin-dependent Kinase 20)^91^ with a Kd value of 8.9 ± 1.6 μM within 30 days from target selection and after only synthesizing 7 compounds. This compound was the first small molecule targeting CDK20 at that time, and this work is the first demonstration of AF2’s successful application in the early drug discovery process.

Weng et al.^92^ applied AF2 to predict the 3D structure of WSB1 (SOCS-box-containing WD-40 protein), a new potential anticancer target^93–95^ with 3D structural information not available. The predicted structure was then optimized by molecular dynamics simulations. The optimized 3D structure of WSB1 was taken as the receptor structure to perform molecular docking to screen for WSB1 inhibitors. Finally, they obtained a number of potential active compounds. Among these compounds, G490-0341 displayed the best stable structure and deserved further research and development.

Liang et al.^96^ identified JMJD8^97–99^ as a novel oncogene correlated with immunosuppression and DNA repair by bioinformatics analysis. Then they used AF2 to predict the 3D structure of JMJD8 and performed virtual screening to retrieve JMJD8 inhibitors. Liu et al.^100^ proposed a multi-target drug discovery method and applied this method to drug discovery of therapeutic hypothermia.^101^ In this study, they first predicted the structure for all related protein targets by using AF2 and RoseTTAFold. After that, they applied molecular docking to estimate the interaction between proteins and drugs, and determined optimal single drugs or drug combinations. Considering the differences in the weights of different protein targets, the approach could refrain from inhibiting beneficial proteins effectively while inhibiting harmful proteins.

Except for the above examples, some researches also showed that the side chain quality modeled by AF2 is not good enough for drug discovery, and some recent studies also found that the docking test based on AF2-predicted structures showed weak enrichment performance.^102,103^

Other researches with AF2 applied in drug discovery include the literature.^104–106^

#### Protein design

Design of proteins means creating novel proteins with desired structures and functions. De novo protein design is a longstanding fundamental goal of synthetic biology.^107–109^ It is a complex and challenging task, which is mainly hindered by the difficulty in reliable prediction of protein 3D structures from amino acid sequences. AF2 as well as other machine learning algorithms (such as RoseTTAFold and recent language models^49,50^) likely removes this obstacle. It is no exaggeration to say that with AF2 prediction, we will step into a new era of protein design. Some typical protein design examples by using AF2 are given as follows.

Jendrusch et al^110^ developed a computational framework for de novo protein design that embeds AF2 as an oracle within an optimizable design process. This is an adaptable framework for protein design through sequence optimization utilizing evolutionary algorithms. It extends previous studies towards protein design by leveraging structure predictors.^111,112^ The integrity of the structures predicted is validated and confirmed by standard ab initio folding, protein structure analysis methods, and rigorous all-atom molecular dynamics simulations. They also showed a potential application of their method in designing de novo protein monomers, dimers and oligomers, as well as protein binders for target proteins and proteins which change conformation upon complex formation.

Goverde et al.^113^ designed a pipeline for de novo protein design based on AF2. In the beginning, they just inverted the AF2 model, utilizing a loss function and the prediction weight set to bias the generated sequences for the objective of adopting a target fold. However, as observed in the protein surface’s hydrophilic versus hydrophobic patterning, the approach does not seem to fully capture basic principles of de novo protein design. Then they made modifications to their pipeline system with minimal post-design intervention, and conducted in vitro validation, which demonstrated that some designs were folded and stable in solution in the condition of high melting temperatures. Overall, the revised pipeline generated viable sequences as assessed experimental characterization, showing the possibility of contributing to solving outstanding challenges in the field of de novo protein design.

Other interesting studies with AF2 assisting protein design include the literature.^114–117^ It is also necessary to mention that, relative to AF2, RoseTTAFold has more applications in protein design, largely due to Baker’s groundbreaking work.^118–120^

#### Target prediction

Target prediction, including on-target and off-target identifications, are important not only for understanding physiological and pathological processes, but also for identifying novel drug targets and evaluating selectivity of drugs. Experimental approaches to target identification, such as various activity-based protein profiling (ABPP)-based methods,^121–124^ are often expensive and time consuming. Computer-aided target prediction may help narrow the scope of target identification, which is often based on protein-ligand docking, usually called inverse docking. Previously, the inverse docking faces a challenge of lacking 3D structures of all possible protein targets. The AF2 structures provide an unprecedented opportunity to develop feasible target prediction methods.

Wang et al.^125^ utilized the AF2 structures to construct the first pocket library for all the proteins in the human proteome, called the CavitySpace database. CavitySpace can be applied to identify novel targets for known drugs in drug repurposing or side effect researches. This database can be easily used to the target prediction by inverse docking. The building workflow of database is as follows: they collected 23,391 human proteins from AlphaFold protein structure database and 6956 human reference proteins from PDB. Then, they applied CAVITY, a tool developed by the same research group to detect all the possible cavities on protein surfaces,^126^ to identify all the potential cavities on protein surfaces. The CavitySpace database is freely available at http://www.pkumdl.cn:8000/cavityspace/.

There are also other related studies applied AF2 for target prediction.^127–129^

#### Protein function prediction

Currently, there are still many proteins whose functions are not known or poorly understood. Since 3D structures of proteins completely determine their functionality, this characteristic can be utilized to establish data-driven prediction models of protein function. Nevertheless, the insufficient number of available protein structures severely limits the performance of these models. The structures predicted by AF2 have provided a promising solution towards this problem, and are expected to improve the performance of these models via increasing the amount of training samples.

Ma et al.^130^ recently conducted a comprehensive study to investigate whether AF2-predicted structures could enhance the protein function prediction performance. In this study, they proposed a state-of-the-art structure-based protein function prediction approach and constructed a new benchmark data set. After that, they evaluated whether the performance of the protein function prediction model could be improved by putting additional protein structures predicted by AF2 into the training data set. They further compared the performance differences between two models separately trained with structures predicted by AF2 only and with real protein structures only. Their results demonstrated that protein function prediction models based on structures could benefit from virtual training data composed of structures predicted by AF2. Even, the model trained only using the structures predicted by AF2 achieved comparable performances to the model based on real protein structures, which are solved through experiments. This indicates that the structures predicted by AF2 were almost equally effective in protein function prediction.

Hu et al.^131^ explored the utility of the Protein Language Models (PLMs) module in AF2, Evoformer, in protein function prediction, and particularly compared the performance of evolution-based & evolution-free protein language models as protein function predictors. They showed that evolution-based PLMs performed better than evolution-free models only in the structure prediction tasks, but in general, were worse than evolution-free models in most function prediction tasks. Consistent with structure prediction, evolution-based PLMs are also sensitive to the amount of MSAs when predicting protein function.

Interpretable and compact structural feature representations are important for accurate prediction of protein properties and function. In a recent study, Rappoport and Jinich^132^ constructed and evaluated 3D feature representations of protein structures using space-filling curves, in which AF2 predicted protein structures were used. In this study, two enzyme substrate predictions were used as case studies: the S-adenosylmethionine dependent methyltransferases (SAM-MTases) and the short-chain dehydrogenase/reductases (SDRs). As their results demonstrate, enzymatic function could be predicted from feature representations on the basis of the 3D structures of SAM-MTAses and SDRs with good accuracy.

By searching proteins that contain Zα domain (experimentally validated Z-DNA/Z-RNA^133^ binding protein domain) from AF2 predicted structure database, Bartas et al.^134^ identified 185 proteins with a putative Zα domain, which may bind to Z-DNA/Z-RNA and play an important role in a variety of cellular processes.

There are also other interesting researches related to protein function prediction involving AF2.^135,136^

#### Protein–protein interaction

Protein-protein interaction (PPI) refers to the process in which two or more protein molecules form a protein complex through non-covalent bonds.^137,138^ A majority of proteins need to recruit other proteins through PPI to form protein complexes to perform their functions. Understanding the structure of interacting proteins is a fundamental step towards revealing the protein function and mechanism. However, there is lack of computational tools that can produce accurate structures of protein complexes. The emergence of AF2 can be greatly conducive to this area.

Evans et al.^139^ extended AF2 to the prediction of multiple-chain complex, and the system was named as AlphaFold-Multimer. On a benchmark data set of 17 heterodimer proteins without templates, they achieved at least medium accuracy on 14 targets and high accuracy on 6 targets. They also predicted structures for a large data set containing more than 4,000 recent protein complexes, from which they scored all the non-redundant interfaces with low template identity from these protein complexes. For heteromeric interfaces, they successfully predicted the interface in 67% of the cases, and 23% of the cases were predicted with high accuracy. For homomeric interfaces they effectively predicted the interface in 69% of cases, and produced high accuracy predictions in 34% of cases. All these results demonstrated superior performance compared to existing approaches. The AlphaFold2-multimers has now been used to predict protein-protein complex structures. For example, Gómez-Marín et al.^140^ applied AlphaFold-multimer for the prediction of PHF14-HMG20A complex models. Ivanov et al.^141^ also applied AlphaFold-multimer to predict the homodimers structure of CYP102A1.

Recently, Bryant et al.^142^ applied AF2 to predict heterodimeric protein complexes. In this work, they explored the docking effect by using the AF2 pipeline combined with different input MSAs, which is for studying the relationship between the output model quality and these inputs. Through scoring multiple PPI models with a predicted DockQ score (pDockQ), they could distinguish from incorrect models with high confidence acceptable (pDockQ > = 0.23). They concluded that AF2-based docking outperformed another docking method.^143^

Yin et al.^144^ examined the performance of AF2 in predicting structures of protein complexes from amino acid sequence. They used 152 diverse heterodimeric protein complexes to form a benchmark test data set. In this test, 43% of cases that had near-native models were produced as top ranking prediction results by AF2, substantially outperforming the performance of unbound protein–protein docking method (9%). To examine the effect of AlphaFold_Multimer in predicting antibody–antigen interaction, the authors used a set of antibody-antigen structures, which were released recently. The testing results confirmed a low success rate for the modeling of antibody–antigen complexes. They further observed that, via the algorithm, T cell receptor–antigen complexes are similarly not accurately modeled. These findings demonstrate that AF2 faces challenges in handling the adaptive immune recognition. Gao et al.^145^ developed an AF2-based system called AF2Complex, which can predict direct physical interactions in multimeric proteins. Contrary to normal approaches, paired MSAs are not necessary for AF2Complex. It improves significantly over AlpahFold-Multimer and reaches higher accuracy compared to some complex protein-protein docking approaches. Moreover, the authors introduced metrics used for direct protein-protein interactions prediction between arbitrary pairs of proteins and validate AF2Complex on the E. coli proteome as well as some challenging benchmark sets.

In addition to PPI, AF2 can also been used in the prediction of peptide-protein interactions. For example, Tsaban et al.^146^ suggested an AF2-based strategy to model peptide-protein complex which did not require MSA information for the peptide partner. In this way, binding-induced conformational changes of the receptor could be handled. The outcomes demonstrate that AF2 could be expected to provide structural insight into a broad range of peptide-protein complexes.

For some similar investigations related to protein-protein interaction or protein-peptide interaction by using AF2, readers can see the literature.^147–153^

#### Biological mechanism of action

Exploring biological mechanism of action is often complicated and remains a challenge. Studies of biological mechanism of action include many aspects, such as drug-target interaction mode, mechanism of biological enzyme catalysis, and so on.

In silico molecular docking methods have been broadly applied to the prediction of drug-target interaction. Nevertheless, this kind of methods strongly rely on existing protein structures. AF2 provides alternative approach to retrieval of accurate protein structures. Wong et al.^102^ combined molecular docking simulations with AF2 for protein-ligand interactions prediction. They successfully predicted the interactions between 296 proteins spanning the essential proteome of Escherichia coli, and 218 active antibacterial compounds and 100 inactive compounds, respectively. They measured the enzymatic activity of 12 essential proteins which were treated with each antibacterial compound to benchmark the performance of the model. This research suggests that advanced approaches in modeling protein–ligand interactions, especially utilizing methods based on machine learning, are needed to better leverage AF2 for mechanism of action studies as well as drug discovery.

Lactate oxidation with NAD+ as electron acceptor^154^ is a highly endergonic reaction. Some anaerobic bacteria conquer the energetic barrier through electron bifurcation/confurcation (FBEB/FBEC) based on flavin utilizing a lactate dehydrogenase (Ldh) combining with EtfA and EtfB, which are the electron-transferring proteins. However, the mechanism of action is poor understood. In a recent study, Kayastha et al.^155^ utilized AF2 calculations and obtained a plausible new B (bifurcation-connected) state, which allows electron to transmit between the shuttle FADs and EtfAB base. Based on the findings, they put forward an integrated catalytic mechanism of the FBEC process.

Post-transcriptional RNA editing regulates the expression of gene in a condition-dependent manner, which mechanism remains unclear. In a recent study, Kimura et al.^156^ characterized the C-to-Ψ editing mechanisms. They showed that TrcP mediates the stepwise editing of C-to-U followed by the conversion of U to Ψ. The structure modeling based on AF2 revealed a distinct long helical domain within TrcP which possibly binds and orients the substrate tRNA during both reactions, The findings suggest that TrcP mediated C-to-Ψ editing depends on a substrate channeling mechanism. These discoveries offer mechanistic views into an RNA editing process which could possibly stimulate environmental adaptation.

Liang et al.^157^ explored the substrate determinants and recognition mechanism of separase, which is a giant cysteine protease. In budding yeast, they identified a conserved motif downstream of the cleavage site. Using AF2 and molecular dynamics simulations, they discovered that in a conserved cleft near the binding groove of separase’s inhibitor securin, the motif is recognized by separase. The binding is mutually exclusive and requires separase’s conformation changes. Their research could let scientists get a deeper understanding of mechanism of substrate recognition and activation of separase.

Lorenz et al.^158^ applied AF2 to predict the structure of selected KRAB^159^ domains. They discovered an evolutionary conserved L-shaped body of two α-helices in all the domains of KRAB. It is changed into a typical spatial arrangement especially for mKRAB-AB after the replacement of amino acid and together with a third helix provided by mKRAB-B. This provides basic insights of how KRAB form complex with TRIM28. McMullen et al.^160^ found from yeast that EKP-GCSF and GCSF exhibits similar binding to its receptor GCSF-R. Similarly, to study the structural effects of EKP^161,162^ on GCSF, they applied computational modeling using AF2 in conjunction with molecular dynamics simulations. Computational modeling shows that EKP does not change the structural behavior of GCSF, which demonstrates that EKP does not hinder receptor binding. Furthermore, the initial conformation of EKP-GCSF from AF2 shows that the EKP which is around GCSF might provide evidence of the thermal-protectivity of EKP on GCSF.

There are also other studies pertaining to biological mechanism of action.^163–176^

#### Other applications

In addition to the application areas mentioned above, AF2 prediction could also be applied to some other fields, such as protein evolution,^177–180^ rare disease treatment studies,^181^ effects of mutation on treatment,^182–187^ vaccine design,^188–190^ and so on. For example, Tang et al.^180^ investigated the relationship between organism evolution and protein evolution based on the structures of proteomes from 48 organisms predicted by AF2. They found some interesting phenomena, including: (1) constituent proteins of organisms with higher complexity would have larger gyration radii, higher coil fractions as well as slower vibrations, and (2) higher degree of functional specialization of proteins is associated with higher degree of organismal complexity. This research brings new views about how the proteins’ functionality diversity increases, and how the dimensionality of the manifold of protein dynamics decreases in the process of evolution. Sebastiano et al.^181^ found that protein structures predicted by AF2 have a potential to assist rare disease treatment studies. In this investigation, the authors focused on Alsin, a protein responsible for rare motor neuron diseases. With the AF2 predicted protein structures, they evaluated the flexibility profile of Alsin and its mutants, and models of dimeric/tetrameric Alsin responsible for its physiological action. They concluded that efforts of drug discovery targeting Alsin-involving diseases should be pursued. Yang et al.^184^ applied AF2 to predict the S, N, and M proteins’ structures of the Omicron variant of SARS-CoV-2. They analyzed how the S protein and its parts, S1 RBD and NTD, have been affected by the mutations in detail, and also how the current SARS-CoV-2 vaccines and treatments would be affected by these mutations. Zeng et al.^188^ utilized AF2 to design a hemagglutinin stem vaccine “B60-Stem-8070”. This vaccine showed better performance compared with the original hemagglutinin stem antigen.

### Limitations of current AF2 prediction

The invention of AF2 is a game-changer event in structural biology. It has reformed the field of protein structure prediction by utilizing sequence information to model protein folds quickly with atomic-level accuracy. However, current AF2 was trained on protein structures from the Protein Data Bank in which X-ray crystallographic structures dominate. Therefore, it is best considered as a predictor of the structured state under experimental conditions where a protein is likely to crystallize, other than a predictor of the lowest free-energy state under physiological conditions. This together with some inherent limitations in methods and techniques limits applications of AF2 predictions in many aspects, which are summarized as follows.

#### The protein dynamics

Protein dynamics is a very important research area.^191–197^ The protein structure predicted by AF2 is a static state. However, proteins are very dynamic with multiple states. Many important physiological and pathological proteins (such as ion channel proteins) have very subtle conformational changes under different active states, and will also show ever-changing spatial configurations due to their combination with various other proteins inside and outside the cell. At this point, AF2 often gives a single optimal solution, which is difficult to cover the conformational diversity of proteins. However, this does not mean that it is not possible to understand protein dynamics with AF2. According to several recent studies,^198–201^ AF2 can still be used for some analysis of protein dynamics. For example, Del Alamo et al.^199^ recently presented a method to drive AF2 for sampling alternative conformations of topologically diverse transporters as well as the G-protein-coupled receptors that do not exist in the training dat set of AF2. Nevertheless, the exploration of the conformational space is in part a by-product of low sequence information which is provided for inference.

There are also other studies pointing out that AF2’s weak performance in identifying conformational ambiguity.^202^ Additionally, AF2 could hardly be used for the structure prediction of a protein with multiple domains, such as a transmembrane receptor with a large extracellular domain.^203^ There is still a demand of new deep learning methods to be designed for the prediction of ensembles of biophysically correlated states.

#### Structures for disordered regions of proteins

The AF2 database consists of highly accurate predictions for the folded part of a large number of proteins. Nevertheless, AF2 does not well in predicting structures of proteins, in cases fewer sequences are available for alignment, and regions that are natively unfolded or disordered regions, for example, loops. The loop structures are relatively stable in crystals, but are very flexible in solutions. Although many approaches have been tried,^204–207^ it is difficult for existing methods to predict the morphology, dynamics and interactions of disordered regions of proteins in solutions.

#### Structures of proteins in complex with small molecules or other proteins

It has been well known that small molecule ligands or proteins may induce a protein to undergo conformational changes. The most representative example is allosteric modulators,^208^ which refers to small molecules or peptides that bind to a site of an enzyme protein different from its endogenous ligand binding site to cause conformational changes, thereby changing the activity of the enzyme. Besides allosteric modulators, plenty of orthosteric ligands, which bind to the identical site as the endogenous ligand, can also induce conformational changes. Nevertheless, AF2 is not designed to determine how proteins change their shape in the presence of other interacting ligands or proteins.

#### Structures of proteins with point mutations

Point mutations are frequently encountered in proteins, particularly pathological state. Understanding the effect of missense mutations on protein structure may help unveil their biological or pathological mechanism. Even though AF2 could predict wild-type (WT) structures, it likely performs poorly in predicting the effect of missense mutations on the proteins’ 3D structures. Although there are researches showing that AF2 could predict the phenotypic effect of missense mutations,^209,210^ it was also observed that the performance of missense mutations prediction is not good in other studies,^211^ and there were only weak or no correlations between the output metrics of AF2 and changes in protein stability or functionality.^212^

#### Structures of proteins with post-translational modifications

Post-translational modifications,^213–215^ such as phosphorylation,^216^ methylation,^217^ acetylation,^218^ and glycosylation,^219^ are common in proteins. These post-translational modifications may lead to conformational changes in protein structures.^220^ For example, phosphorylation of inactive kinases in their active loop often results in a large conformational change, and eventually activate the kinases. However, AF2 can predict protein structures only based on their amino acid sequence, and post-translational modifications of residues cannot be recognized. Therefore, the conformational changes due to post-translational modifications cannot be predicted with current AF2.

#### Prediction of orphan proteins and artificially designed proteins

In addition to the above-mentioned limitations, AF2 and other computational systems that use DL and the information of co-evolutionary relationships encoded in MSAs also face challenges in prediction of orphan proteins and artificially designed proteins because an MSA cannot be generated. Recently Chowdhury et al.^51^ developed an end-to-end deep neural network model, namely differentiable recurrent geometric network (RGN) model, in which a protein language model (AminoBERT) based on the Bidirectional Encoder Representations from Transformers (BERT)^221^ was used to learn latent structural information from unaligned proteins. Language models were firstly introduced to extract semantic information from words. The RGN model showed better performance on orphan proteins than AF2, while significantly reducing the computing time by up to 10^6^-folds. These results demonstrate the theoretical and practical strengths of protein language models in structure prediction compared with MSAs.

#### Limitations in methods and techniques

We finally have to mention that AF2 itself has some limitations in methods and techniques. For example, (i) deep learning models have low interpretability currently; (ii) the AF2 structural prediction is based on the data of MSA, i.e., a large number of evolutionarily related sequences is needed for the structure predictions, which might cause side effects such as comparably slower prediction speed. As a comparison, language models (such as ESMfold^49^ and RGN^50^) enable end-to-end protein structure prediction directly from amino acid sequences with high speed and accuracy.

## Concluding remarks

The excellent performance of AF2 in predicting protein structure together with the release of structures of more than 200 million proteins predicted by AF2 is reshaping structural biology. AF2 will certainly have a significant impact on researches that need protein structure information, and could be applied in many fields such as drug discovery, protein design, target prediction, protein function prediction, PPI, biological mechanism of action, and others, in addition to experimental structural biology. Despite just a very short time since AF2 was developed, we have already witnessed a number of successful applications. We believe that, as time goes on, more applications or new application fields will be developed, for example, design of protein machines with complex or specific functions, design of new organisms, and disease diagnosis. Even so, AF2 prediction is not a panacea and there are many issues still needing to be solved, including protein dynamics, structures of disordered regions of proteins, structures of mutants, structures of protein-ligand complexes, structures of proteins with post-translational modifications, and so on. With the further development of AI algorithm, ever-increasing data, and computing power, it is expected that more surprises will surely come to us in future.

Finally, it is necessary to mention that, during the revision of this article, the new CASP competition, CASP15, is over. Different from previous CASPs, which took protein structure prediction as the main track, CASP 15 also paid attention to predicting protein complex and RNA structures. This is consistent with the CASP’s style or philosophy that keeping pace with the times, which means that protein complex structures and RNA structures might be the new focuses in the “post AlphaFold era”. Another noteworthy point is that DeepMind did not take part in CASP15, which reasons are unclear. Nevertheless, all teams that have achieved better results have more or less used AF2 algorithms or AF2 predicted structures, which implies that AF2 invisibly won CASP15 again, further highlighting the great influence of AF2 in structural biology. Overall, we look forward to new breakthroughs of AI in structural biology, and more application achievements by using AF2 in the future.
